# Visualizing Three-Qubit Entanglement

**DOI:** 10.3390/e27080800

**Published:** 2025-07-27

**Authors:** Alfred Benedito, Germán Sierra

**Affiliations:** 1 Instituto de Física Teórica, UAM-CSIC, Universidad Autónoma de Madrid, 28049 Madrid, Spain; 2 Kavli Institute for Theoretical Physics, University of California, Santa Barbara, CA 93106, USA

**Keywords:** quantum entanglement, entanglement polytope, entanglement invariants, three-qubit state, canonical form, tamgle, entanglement classes

## Abstract

We present a graphical framework to represent entanglement in three-qubit states. The geometry associated with each *entanglement class* and *type* is analyzed, revealing distinct structural features. We explore the connection between this geometric perspective and the tangle, deriving bounds that depend on the entanglement class. Based on these insights, we conjecture a purely geometric expression for both the tangle and Cayley’s hyperdeterminant for non-generic states. As an application, we analyze the energy eigenstates of physical Hamiltonians, identifying the sufficient conditions for *genuine tripartite* entanglement to be robust under symmetry-breaking perturbations and level repulsion effects.

## 1. Introduction

Entanglement is a consequence of the superposition principle, where quantum states cannot be written in product form on any local basis [1]. Although their existence was first pointed out by Einstein, Podolski and Rosen in 1935 [2], it was not until the late 1990s and early 2000s that the study and classification of entanglement in systems of more than two qubits piqued the interest of physicists due to the realization that they could be used as a resource in information processing and communication. This is because entanglement differs from classical correlations even if one uses local hidden variables [3].

**Figure 1:**
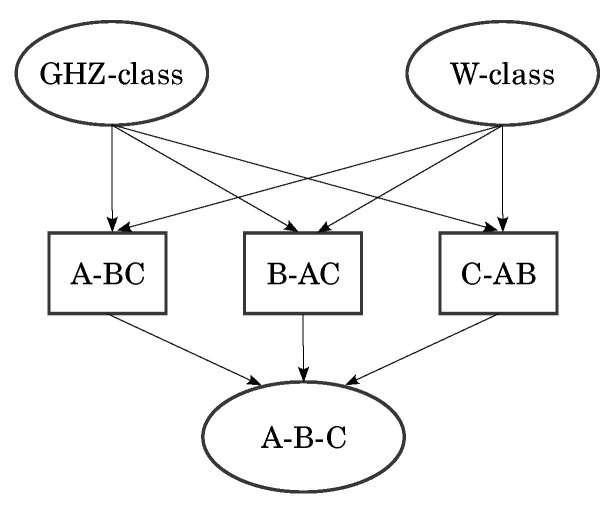
Entanglement classes (from [13]).

The GHZ state [4,5] sparked interest in the aforementioned classification, with early attempts revolving around the study of the orbits of $U{\left(2\right)}^{⊗n}$ [6] but later moving to the modern paradigm of *entanglement measures* [7] such as Benett’s *entanglement of formation* [8]. This measure was later extended to any two-qubit mixed state [9,10] through a quantity known as *the concurrence* C. With it, it was shown that, if one has a system of three qubits *A*, *B* and *C*, then there is a trade-off between *A*’s entanglement with *B* and with *C* [11]. In other words, the sharing of entanglement is restricted. This is a core difference between classical and quantum correlations, measured by *the tangle* τ. Moreover, Wooters, Coffman and Kundu conjectured that such a relation should also exist for systems of more qubits (known as the CKW conjecture, later proven to be true by Osborne and Verstraete [12]). This showed that there were two inequivalent ways of entangling all three qubits in a pure three-qubit state, allowing for the classification of pure three-qubit states [13] based on defining entanglement classes as sets of states that map onto themselves under invertible SLOCC (these methods can be used to classify four-qubit states as well [14]). They identified six different entanglement classes (see Figure 1), fully characterized by four parameters (see Table 1).

From the group orbit analysis [6], it was found that the number of entanglement-invariant parameters had to be five for three-qubit states, but so far, only four have been used. This points towards the existence of a further structure yet to be found within these classes. This structure was understood by means of a *Generalized Schmidt*/canonical decomposition (CD) for three-qubit states [15]. The CD showed that there is a *canonical form*, unique for all states related by Local Unitaries (LUs), that uses only five of the eight basis elements. Depending on which basis elements have null coefficients, one could identify different sub-classes (or *types*).

This classification is quite difficult to visualize, because it depends on the five entanglement invariants ${\left\{J_{l}\right\}}_{l=1}^{5}$ (which are hard to relate to physical observables). For one-qubit states, a typical graphical representation is the Bloch sphere, $S^{2}$, representing the expectation values of spin observables. For two qubits, Mosseri and Dandoloff [16] reinterpreted this Bloch sphere map as fibrating $S^{3}$ with $S^{1}$: a Hopf fibration. This way, the generalization of the Bloch sphere follows immediately. For three qubits, one can do a similar construction [17], but the fundamental difference (for our interests) is that the construction is then sensitive to the entanglement between *A* and BC but it says nothing about the entanglement between *B* and *C*. Mosseri proposed for this case to instead use the three Bloch-norms $\left(r_{A},r_{B},r_{C}\right)$, which are also entanglement invariants (they belong to a set first found by Sudbery [15,18]). It is these three invariants along with the tangle that will be the main focus of our work.

The main aim of this paper is investigating how three-qubit entanglement can be visualized with physical observables in a geometrical picture. We study how Mosseri’s proposal results in a more physical and geometrical characterization of different entanglement classes and *types* in what we call *Bloch-norm representation*. This naturally leads to a series of bounds between the tangle and the norm of a vector. An important consequence of those results is that we can conjecture a formula for calculating Cayley’s hyperdeterminant [11,19,20,21], which relies purely on geometrical characteristics. This is fairly striking, since contrary to the regular determinant (which can be understood geometrically as a measure of volume), the hyperdeterminant does not have a simple geometric interpretation. The second goal is the application of these geometrical tools to the study of the entanglement present in the energy eigenstates of physical Hamiltonians, where we identify sufficient conditions for *genuine tripartite entanglement* to be robust under perturbations.

This paper is organized as follows. In Section 2 we briefly review the graphical aspect of Mosseri’s proposal. The original work of this paper begins in Section 3, which is devoted to the study of the relation between the Bloch-norm representation and the tangle. We derive bounds for τ depending on the Bloch-norms and geometrically characterize it for states belonging to the GHZ class. Finally, in Section 4, we consider physical systems of three qubits characterized by different Hamiltonians. We study the Bloch-norm properties of their energy eigenstates and characterize the source of their tangle. To do so, we developed a Python v3.13.3 library to automate the analytic computations as much as possible. Finally, Appendix B contains the details of the fibration procedure needed to obtain the bounds of Section 3, and in Appendix C, we provide the exact calculations for the chains in further detail.

## 2. Graphical Representation of Entanglement

Let us first present a summary of the classification of three-qubit states and their tangle. Starting with the concurrence, this is a quantity which measures the entanglement between two bipartitions of a state. In the simplest case, a two-qubit state of components ${\left\{v_{ij}\right\}}_{i,j\in \{0,1\}}$, it reduces to $\propto |v_{00}v_{11}-v_{01}v_{10}|$, which is 0 if the state is separable and >0 if it is entangled. In three-qubit states, there exist three possible bipartitions: $C_{AB}$, $C_{AC}$ and $C_{A(BC)}$. By comparing them, one can find the following:

(1) $$C_{AB}^{2}+C_{AC}^{2}\leq C_{A(BC)}^{2}$$

which motivates the definition of the *tangle*:

(2) $$\tau_{ABC}:=C_{A(BC)}^{2}-\left(C_{AB}^{2}+C_{AC}^{2}\right)$$

This tells us that *A* can be entangled with BC (measured by $C_{A(BC)}$) in an essential way that cannot be described, in general, through a combination of the entanglement of *A* with *B* (measured by $C_{AB}$) and of *A* with *C* (measured by $C_{AC}$). If this is the case, we say that this tripartite entanglement is *genuine*. As stated in the introduction, this is what differentiates the entanglement present in the state $|W\rangle =\frac{1}{\sqrt{3}}\left[|001\rangle +|010\rangle +|100\rangle \right]$ and the state $|\mathrm{GHZ}\rangle =\frac{1}{\sqrt{2}}\left[|000\rangle +|111\rangle \right]$:

- $\tau \left(W\right)=0$. This reflects that W can be written as a superposition of all three possible Bell pairs—$|W\rangle \propto {|\phi_{+}\rangle }_{AB}{|0\rangle }_{C}+{|\phi_{+}\rangle }_{AC}{|0\rangle }_{B}+{|\phi_{+}\rangle }_{BC}{|0\rangle }_{A}$—so its entanglement is fully pair-wise-generated.
- $\tau \left(\mathrm{GHZ}\right)=1$. This reflects that for $|\mathrm{GHZ}\rangle$ no pair-wise decomposition exists. In fact, $C_{IK}=0$, ∀I,K∈{A,B,C} and $C_{I(\bar{I})}=1$, ∀I, so its tripartite entanglement is *genuine*.

We now present the CD:

(3) $$\begin{array}{ccc} |\psi \rangle & = & \sum_{i,j,k\in \{0,1\}}\left(t_{ijk}{|i\rangle }_{A}{|j\rangle }_{B}{|k\rangle }_{C}\right)\\ |\psi \rangle & \overset{\mathrm{CD}}{\to } & |\lambda_{0},\vec{\lambda },\lambda_{4};\varphi \rangle :=\left[\lambda_{0}|000\rangle +\lambda_{1}e^{i\varphi }|100\rangle +\lambda_{2}|101\rangle +\lambda_{3}|110\rangle +\lambda_{4}|111\rangle \right]; \end{array}$$

where

(4) $$\lambda_{j}\in \left[0,1\right]\forall j;\;\sum_{j=0}^{4}\lambda_{j}^{2}=1;\;\varphi \in \left[0,\pi \right].$$

**Figure 2:**
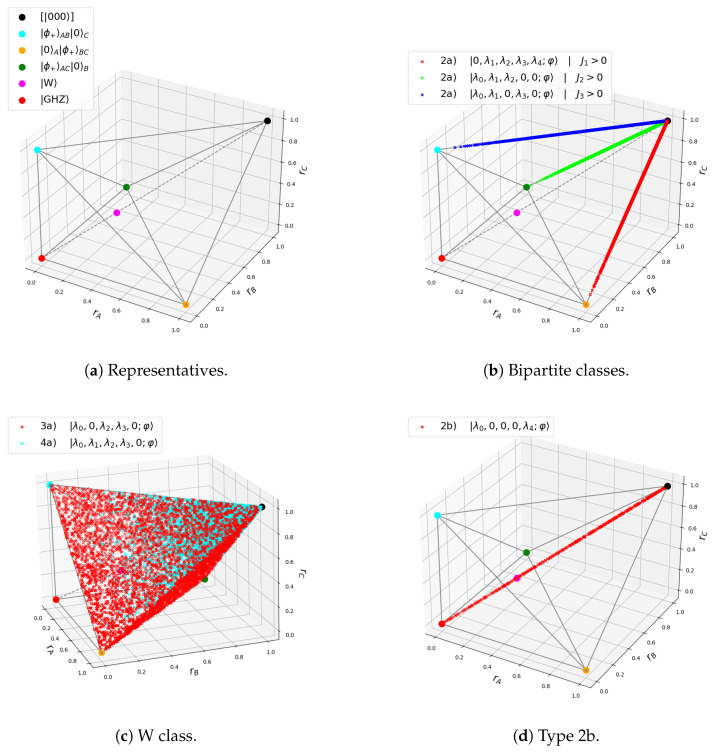

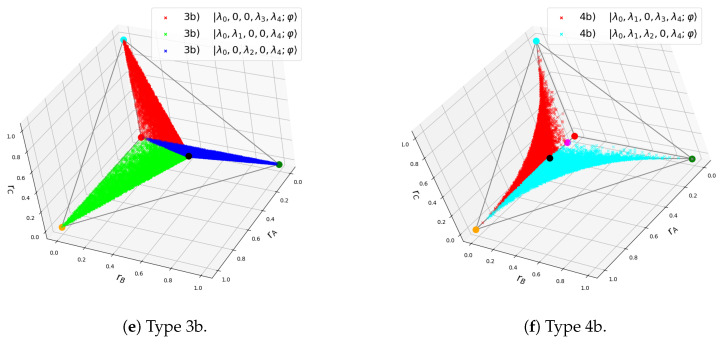
Geometrical representations of the *types* of states.

The λ parameters can be used to calculate ${\left\{J_{l}\right\}}_{l=1}^{5}$ [15]. Finally, we introduce Mosseri’s Bloch-norms: given any *n*-qubit state one can compute *n* different one-qubit reduced density matrices [22]:

(5) $$\rho =\frac{1}{2}\left(I+\vec{r}\cdot \vec{\sigma }\right);\;\mu_{\pm }\left(\rho \right)=\frac{1\pm r}{2};$$

where $\mu_{\pm }\left(\rho \right)$ are the eigenvalues of ρ. We call the Bloch-norm $r\equiv \left|\vec{r}\right|$, which fulfills r=1 if the reduced state is pure and r<1 if the state is mixed. Any three-qubit state will have three different Bloch-norms. The resulting vector of Bloch-norms $\left(r_{A},r_{B},r_{C}\right)$ is restricted to a unit cube ${\left[0,1\right]}^{3}$, so it allows us to graphically visualize the states. However, the full cube cannot be filled. For any given entanglement class, there is a list of linear inequalities that the eigenvalues of the single-particle reduced density matrices have to obey. These inequalities define a *polytope* (a higher-dimensional polygon) in which the states reside [23]. If the eigenvalues violate the inequality, then the point lies outside the polytope and the state does not belong to the specified entanglement class. These inequalities apply as well to Mosseri’s Bloch-norms. Furthermore, there exists a 1:1 relation between the entanglement entropy of each individual qubit $S_{I}$ (I∈{A,B,C}) and its Bloch-norm $r_{I}$:

(6) $$\begin{array}{cc} S(\rho_{I}) & =\frac{1+r_{I}}{2}\log\frac{2}{1+r_{I}}+\frac{1-r_{I}}{2}\log\frac{2}{1-r_{I}} \end{array}$$

so we can reproduce Table 1 in terms the Bloch-norms and the tangle. This will enable us to provide a geometric viewpoint of the states as points inside the polytope. The three-qubit polytope consists of two tetrahedrons glued at a common base. This particular geometrical figure is known as *triangular bipyramid* [24]. The lower and upper tetrahedrons have vertices {(0,0,0),(1,0,0),(0,1,0),(0,0,1)} and {(1,1,1),(1,0,0),(0,1,0),(0,0,1)}, respectively. The representative states of each entanglement class lie on one of the vertices of the polytope, except the W state which lies at the center of the common base (see Figure 2a).

Finally, we list the detailed entanglement classification for three-qubit states combining both the classes from [13] and the types from [15]. We also include our observations on the different geometrical patterns:

1. **Product state class/Type 1:** This contains all three-qubit states with no entanglement, denoted as $\left[A\text{-}B\text{-}C\right]$. It is the equivalence class of $|000\rangle$ under LUs: $\left[|000\rangle \right]$. All states have $J_{r}=0$∀r and $r_{I}=1\;\forall I$, so all states are mapped to (1,1,1) in the polytope.
2. **Bipartite classes/Type 2a:** States of the form ${|\varphi \rangle }_{I}⊗{|\mathrm{Entangled}\;\mathrm{pair}\rangle }_{\bar{I}}$; that is, $r_{I}=1$ and $r_{I^{\prime }}<1$. These states have $J_{l}=0$ for all *l* but one, which could be $J_{1}$, $J_{2}$ or $J_{3}$. States with $J_{1}>0$ correspond to class $\left[\mathrm{BC}\text{-}A\right]$, $J_{2}>0$ to $\left[B\text{-}\mathrm{AC}\right]$, and $J_{3}>0$ to $\left[C\text{-}\mathrm{AB}\right]$. Each class covers one of the three edges of the upper tetrahedron connected to (1,1,1) (see Figure 2b).
3. **W class:** This includes all states with all three qubits entangled, without genuine tripartite entanglement. They can always be written in the following form:
  (7) $$\begin{array}{c} \sqrt{c}|000\rangle +\sqrt{d}|100\rangle +\sqrt{a}|101\rangle +\sqrt{b}|110\rangle \end{array}$$
  with a,b,c>0 and d≥0 [13]. They can be of two types: **Type 3a** *tri-Bell states* and **Type 4a**. **Type 3a** states lie exclusively on the faces of the upper tetrahedron and have $\lambda_{1}=\lambda_{4}=0$, corresponding to the family with d=0 in (7). The W state belongs to this type, with $\lambda_{0}=\lambda_{2}=\lambda_{3}=1/\sqrt{3}$ and $r_{I}=1/3$, ∀I. **Type 4a** states have $\lambda_{4}=0$, corresponding to the family with d>0 in (7). They are located in the upper tetrahedron, and they accumulate near the (1,1,1) point [25] (see Figure 2c).
4. **GHZ class:** This contains states with genuine tripartite entanglement. There are five types:
  (a) **Type 2b** *generalized GHZ states*. They have $J_{l}=0,\forall l$, except for $J_{4}=\tau /4\Rightarrow \lambda_{j}=0$ for j∈{1,2,3}. The standard GHZ state corresponds to the values $\lambda_{0}=\lambda_{4}=1/\sqrt{2}$. They lie on the central diagonal connecting (0,0,0) and (1,1,1) (see Figure 2d). Notice that for $\lambda_{0}\in \left\{1/\sqrt{3},\sqrt{2/3}\right\}$, they occupy the same point in the polytope as the W state.
  (b) **Type 3b** *extended GHZ states*: They have $\lambda_{i}=\lambda_{j}=0$ for j,k∈{1,2,3} with j≠k, so either $\lambda_{1}=\lambda_{2}=0$, $\lambda_{1}=\lambda_{3}=0$, or $\lambda_{2}=\lambda_{3}=0$. Each one spans a different triangle connecting the main diagonal with any of the three vertices of the face {(1,0,0),(0,1,0),(0,0,1)} (see Figure 2e).
  (c) **Type 4b** states have either $\lambda_{2}=0$ or $\lambda_{3}=0$. They lie in the space between two of the three triangles defined by type 3b. If $\lambda_{2}=0$, they lie between the triangles of type *1-2* and *2-3*, while if $\lambda_{3}=0$, then they lie between triangles of type *2-3* and *1-3*. No states of type 4b lie between *1-3* and *2-3* (see Figure 2f).
  (d) **Type 4c** states have $\lambda_{1}=0$. These populate the polytope without any clear pattern.
  (e) **Type 5** *generic GHZ states:* These have $\lambda_{j}\neq 0$ and $J_{k}\neq 0$, ∀j,k. They may lie anywhere in the polytope.

The proofs of the localization of the states in the polytope can be found in Appendix A. For related recent results, see [26]. Observe that the states belonging to the GHZ class occupy any of the two tetrahedra, but the states not in this class are restricted to the upper tetrahedron. In particular, the state with the lowest value of $\left||(r_{A},r_{B},r_{C})|\right|$, with τ=0, is the state W. These observations points towards a connection between the tangle and the Bloch-norm geometrical picture.

## 3. Geometry of the Tangle

In search of such a connection, we wish to study the relation between the tangle and *R*, where *R* is defined as

(8) $$R:=\sqrt{r_{A}^{2}+r_{B}^{2}+r_{C}^{2}}$$

**Figure 3:**
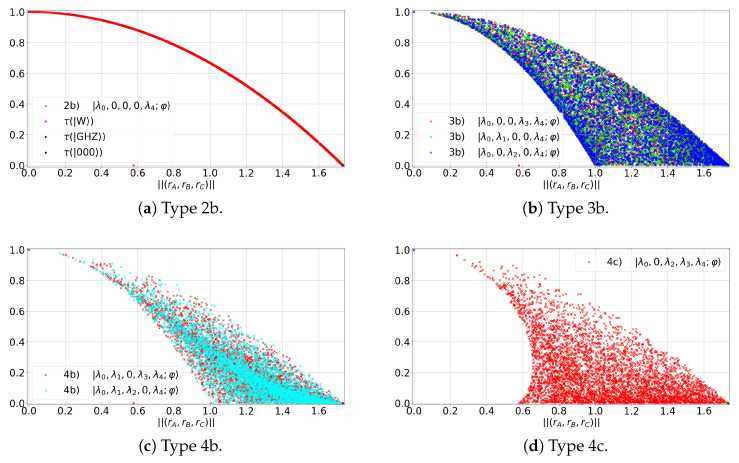
GHZ entanglement class states in the (R,τ) diagram.

For a generic state, by means of the CD (3), one can compute

(9) $$\begin{array}{cc} R^{2} & =3\left(\lambda_{0}^{4}+\lambda_{1}^{4}+\lambda_{2}^{4}+\lambda_{3}^{4}+\lambda_{4}^{4}\right)+6\lambda_{1}\lambda_{2}\lambda_{3}\lambda_{4}\cos\left(\varphi \right)-2\left(\lambda_{0}^{2}\left[\lambda_{2}^{2}+\lambda_{3}^{2}\right]+\lambda_{2}^{2}\lambda_{3}^{2}-\lambda_{1}^{2}\lambda_{4}^{2}\right)\\ & +6\left(\lambda_{1}^{2}\left[+\lambda_{0}^{2}+\lambda_{2}^{2}+\lambda_{3}^{2}\right]+\lambda_{4}^{2}\left[-\lambda_{0}^{2}+\lambda_{2}^{2}+\lambda_{3}^{2}\right]\right) \end{array}$$

where we have not used the normalization condition yet. On the other hand, the tangle can be computed as

(10) $$\begin{array}{c} \tau \left(\psi \right)=4\left|\mathrm{Hdet}\left(t_{ijk}\right)\right|=4\lambda_{0}^{2}\lambda_{4}^{2}=4\lambda_{0}^{2}\left(1-\lambda_{0}^{2}-{\left|\vec{\lambda }\right|}^{2}\right) \end{array}$$

where Hdet is Cayley’s hyperdeterminant [11,19,20,21] and ${\left\{\lambda_{j}\right\}}_{j=0}^{4}$ are the CD parameters. Our aim is to substitute τ into (9) by using (10) and then obtain a function of the form $\tau =\tau (R;\left\{\lambda_{j}\right\})$. For this, we plot (R,τ) for the different types of GHZ states. We find that there are three zones where the states lie (see Figure 3):

- Type 2a states occupy a curve $\tau_{M}\left(R\right)$ (see Figure 3a), maximizing the value of τ for a given value of *R*.
- Type 3b and 4b states occupy a common area (see Figure 3b,c), bounded from above by $\tau_{M}\left(R\right)$ and from below by $\tau_{★}\left(R\right)$.
- Type 4c and 5 states occupy a larger area than the previous cases (see Figure 3d), upper-bounded by $\tau_{M}\left(R\right)$ and lower-bounded by a curve with two branches: $\tau_{↑}\left(R\right)$ and $\tau_{↓}\left(R\right)$.

For type 2a, Equation (9) becomes

(11) $$\tau_{M}\left(R\right)=1-R^{2}/3$$

For types 3b and 4b, one can obtain (see Appendix B)

(12) $$\begin{array}{c} \tau_{★}\left(R\right)=\left\{\begin{array}{cc} 5\tau_{M}\left(R\right)-4\sqrt{\tau_{M}\left(R\right)} & \mathrm{if}\;R\overset{<}{∼}0.56\\ 1-R^{2} & \mathrm{if}\;0.56\overset{<}{∼}R\leq 1\\ 0 & \;\mathrm{otherwise} \end{array}\right. \end{array}$$

where $\tau_{★}\left(R\right)\leq \tau \left(R\right)\leq \tau_{M}\left(R\right)$. For types 4c and 5, the two branches are

(13) $$\begin{array}{cc} \tau_{↑}\left(R\right)=\left(\frac{17}{49}-\frac{5}{21}R^{2}\right)-\frac{32}{147}\sqrt{9-21R^{2}}\; & \;\tau_{↓}\left(R\right)=\frac{1}{4}\left(1-\sqrt{\frac{\left(R_{W}+R_{★}\right)\left(R_{★}-R\right)}{R_{★}^{2}-R_{W}^{2}}}\right) \end{array}$$

where $R_{W}=1/\sqrt{3}$ and $R_{★}=\sqrt{3/7}$, with the following bounding conditions: $\tau \geq \tau_{↑}\left(R\right)$ for $R\leq R_{W}$; either $\tau \geq \tau_{↑}\left(R\right)$ or $0\leq \tau \leq \tau_{↓}\left(R\right)$ when $R\in [R_{W},R_{★}]$; and $\tau \in [0,\tau_{M}\left(R\right)]$ when $R>R_{★}$.

Notice the following properties:

1. States of type 2b maximize τ for a given *R* (see Figure 3a). Moreover, they lie on the main diagonal of the polytope (i.e., $d(\vec{r},{\vec{V}}_{\mathrm{line}})=0$; see Figure 2d).
2. States of types 3b, 4b and 4c deviate from the main diagonal (i.e., $d(\vec{r},{\vec{V}}_{\mathrm{line}})>0$) and have $\tau <\tau_{M}\left(R\right)$.

This leads us to the following geometrical ansatz for the tangle:

(14) $$\tau \left(\vec{r}\right)=1-\frac{{\left|\vec{r}\right|}^{2}}{3}-d\left(\vec{r},V_{\mathrm{line}}\right)\cdot F\left(\vec{r}\right)$$

where $|\psi \rangle \in \mathrm{GHZ}$, excluding type 5, and $F(\vec{r})\geq 0$ is a function encoding the geometric asymmetry of one of the three qubits with respect to the other two. As an example, consider states of type 3b: *3b-12* has $r_{C}>r_{A}$ and $r_{B}$ and *3b-23* has $r_{A}>r_{C}$ and $r_{B}$. Consequently,

(15) $$\begin{array}{cc} F\left(\vec{r}\left(\lambda_{1}=\lambda_{2}=0\right)\right)≃2r_{C}\sqrt{\frac{2}{3}}+O\left(r_{C}^{2}\right) & \;F\left(\vec{r}\left(\lambda_{2}=\lambda_{3}=0\right)\right)≃2r_{A}\sqrt{\frac{2}{3}}+O\left(r_{A}^{2}\right) \end{array}$$

with similar expressions for the different states of each type (see (A19) and (A20)).

With ansatz (14), we have taken an *entanglement measure* (the tangle) and given it a geometrical interpretation; a complementary approach would be the opposite process: propose some notion of geometry for the quantum states and then use it to construct an entanglement measure, which is precisely the approach taken in [27,28].

## 4. Study of the Tangle in Three-Qubit Spin Chains

**Figure 4:**
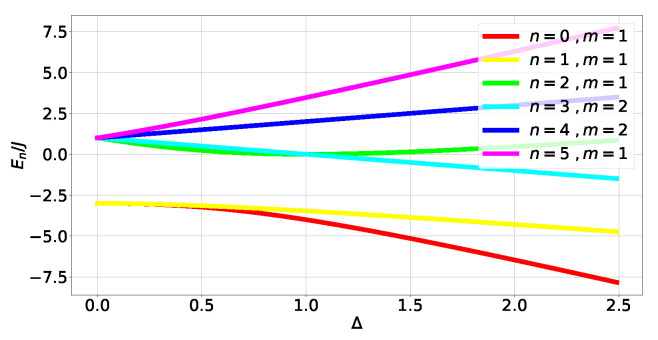
Energy spectrum of $H_{TFIM}$.

**Figure 5:**
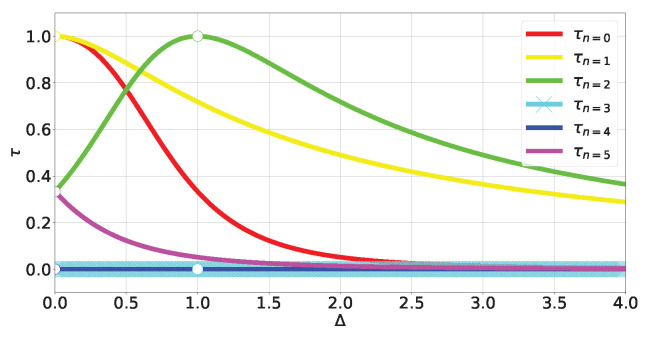
Tangle of TFIM levels (A23) and (A25).

With these tools at hand, we now study the tripartite entanglement present in the energy levels of standard spin-chain Hamiltonians with periodic boundary conditions (PBCs). We begin by studying the Transverse Field Ising Model (TFIM) for three qubits:

(16) $$H_{\mathrm{TFIM}}=-\sum_{j=0}^{2}\left(X_{j}X_{j+1}\right)-\Delta \sum_{j=0}^{2}Z_{j}$$

where Δ≥0. The energy spectrum can be obtained exactly (see (A22) in Appendix C.1 and Figure 4). Obtaining the eigenstates (A23) and (A25)) outside of *level crossings* allows us to calculate the tangle at each energy level:

(17) $$\tau_{n=0,2}=\frac{16f_{n}}{g_{n}^{4}};\;\tau_{n=1,5}=\frac{48f_{n}^{3}}{g_{n}^{4}};\;\tau_{n=3,4}=0;$$

where $f_{n}$ and $g_{n}$ come from components of the eigenstates in the canonical bases (A23) and (A25) and depend on Δ (A28). The (Δ,τ) plot is shown in Figure 5.

**Figure 6:**
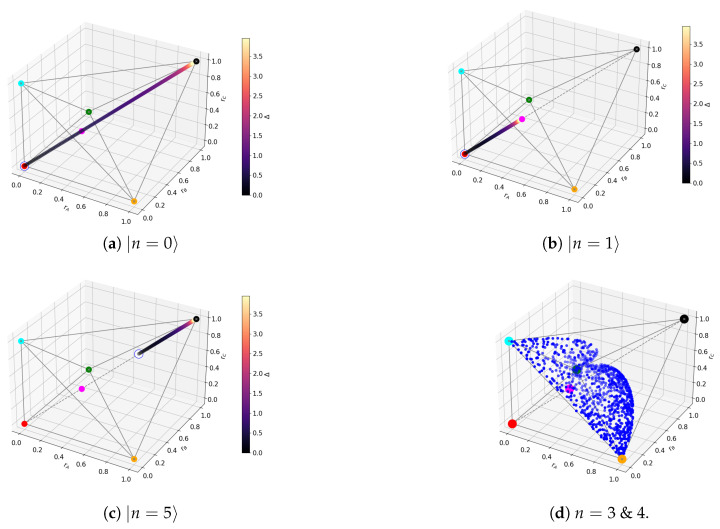
TFIM Bloch-norms out of level crossings. The coloured dots correspond to the representative states as in Figure 2a. The trajectory is plotted for different values of Δ. The white dots mark the discontinuity. For degenerate subspaces, the allowed region is spanned by blue dots.

**Figure 7:**
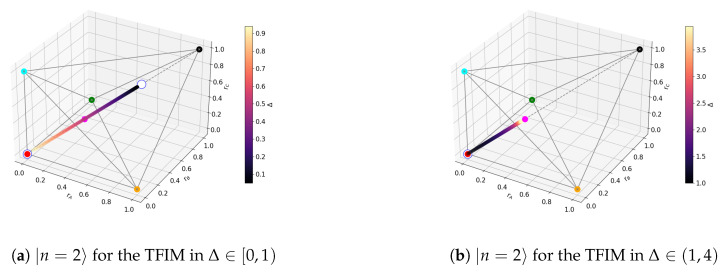
Trajectory for TFIM $|n=2\rangle$.

For non-degenerate subspaces, the Bloch-norms vector (A24) defines a trajectory parametrized by Δ (see Figure 6a–c and Figure 7), while for degenerate subspaces, there is no Δ-dependence and they will span a manifold of dimension greater than 1 (see Figure 6d). This is because the parameters controlling the Bloch-norm values are the weights of the allowed superposition. Notice that when increasing Δ, levels n=1,2 loose their tangle slower than the other levels (see Figure 5). This shows that certain eigenstates have more *robust* tangle than others under changes in the external field Δ.

**Figure 8:**
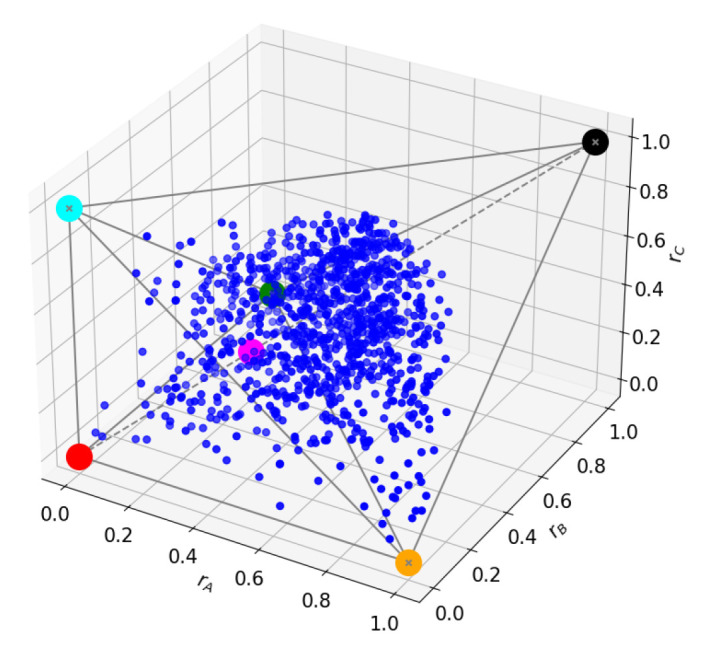
n=2 subspace at Δ=1.

We now turn to the level-crossing points, specifically to Δ=1, where subspaces n=2 and n=3 fuse, corresponding to a degeneracy of m=3 (A39) (see the Bloch-norms in Figure 8, which now span a three-dimensional manifold). The increase in degeneracy allows for new superpositions, changing the tangle of the energy level. This is, in general, the only observable change when considering level crossings: change in the tangle due to an increase in degeneracy. It also makes it less likely that the Bloch-norms will maintain any geometrical pattern because the new superpositions might generate a state that is no longer translation-invariant.

**Figure 9:**
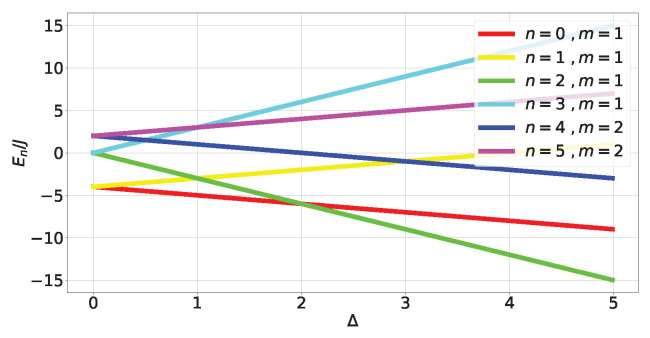
$H_{XX}$ energy spectrum.

Consider the XX chain with a magnetic field Δ:

(18) $$\begin{array}{cc} H_{\mathrm{XX}} & =-\sum_{j=0}^{2}\left(X_{j}X_{j+1}+Y_{j}Y_{j+1}+\Delta Z_{j}\right) \end{array}$$

where Δ≥0, with the exact energy spectrum (A41) shown in Figure 9 (details in Appendix C.2). The Bloch-norms of the non-degenerate levels are all either 1/3·(1,1,1) or (1,1,1) (A43), while for both degenerate levels, the shapes are the same as in Figure 6d.

**Figure 10:**
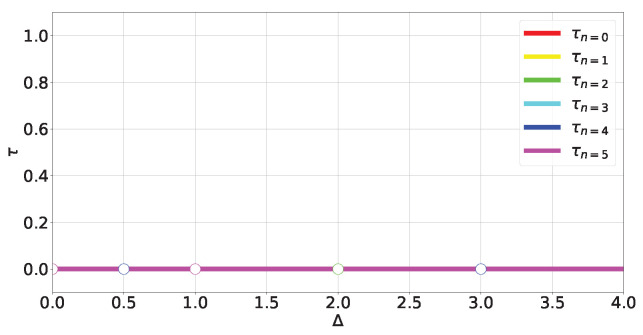
Tangle of XX levels (A42).

Notice that, while in the TFIM the tangle could be intuitively understood as coming from the competition between the two-qubit and one-qubit terms (XX vs. *Z* mediated by Δ), in the XX chain this is no longer the case (see Figure 10). This is because the (XX+YY) terms commute with the *Z* term, producing linear dependence on Δ for the energies and the independence of the eigenstates (and hence the tangle) from Δ.

Furthermore, observe that in the XX chain, the tangle of the energy levels is more *fragile* to perturbations of the Hamiltonian than in the TFIM. The argument is as follows: adding a small perturbation (with parameter ξ) that breaks some symmetry will split the degenerate levels and the whole tangle (which is exclusively due to these degenerate superpositions) will disappear. On the other hand, the tangle for the TFIM states is much more robust under the same procedure, since it does not come from degenerate superpositions. The same argument explains why the tangle generated at level crossings is also fragile: when introducing a small perturbation, the level crossings will generically disappear due to *level repulsion* [29,30] (the phenomenon of level repulsion is known to arise even in classical systems [31,32,33]).

Consider the XXX chain, with the Hamiltonian

(19) $$\begin{array}{cc} H_{\mathrm{XXX}}= & \sum_{j=0}^{2}\left(X_{j}X_{j+1}+Y_{j}Y_{j+1}+\Delta Z_{j}Z_{j+1}\right) \end{array}$$

where Δ∈R. The key difference between the XX and XXX chains is the increase in degeneracies: all levels are degenerate (see (A46) in Appendix C.3). We can find, in general, a non-vanishing tangle:

(20) $$\begin{array}{c} \tau_{n=0}=4\beta^{2}\left(1-\beta^{2}\right)\;\;\tau_{n=1}=\frac{4}{3}\beta^{2}\left(1-\beta^{2}\right)\\ \tau_{n=2}=\frac{4}{9}|{\left(S_{\alpha \beta }S_{\gamma \delta }-\left[\eta_{\beta \alpha }\eta_{\delta \gamma }-\eta_{\alpha \beta }\eta_{\gamma \delta }\right]\right)}^{2}-4S_{\alpha \beta }S_{\gamma \delta }\eta_{\alpha \beta }\eta_{\gamma \delta }| \end{array}$$

where $S_{\alpha \beta }=\left(\alpha +\beta \right)$, $\eta_{\alpha \beta }=\alpha \exp\left(i2\pi /3\right)+\beta \exp\left(-i2\pi /3\right)$, and $\eta_{\beta \alpha }=\alpha \exp\left(-i2\pi /3\right)$ + $\beta \exp\left(i2\pi /3\right)$. Notice that, just as in the XX chain, the tangle here will be fragile under any small perturbation of the Hamiltonian.

**Figure 11:**
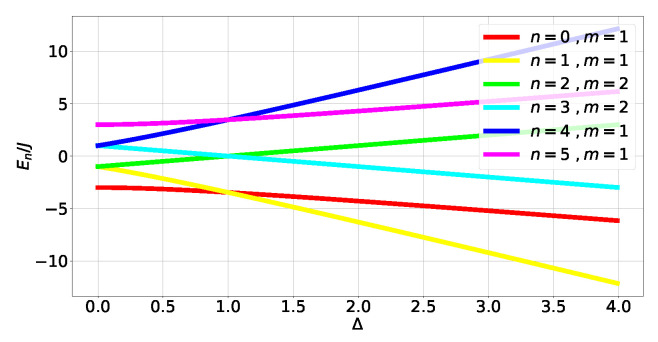
$H_{XZX}$ energy spectrum.

**Figure 12:**
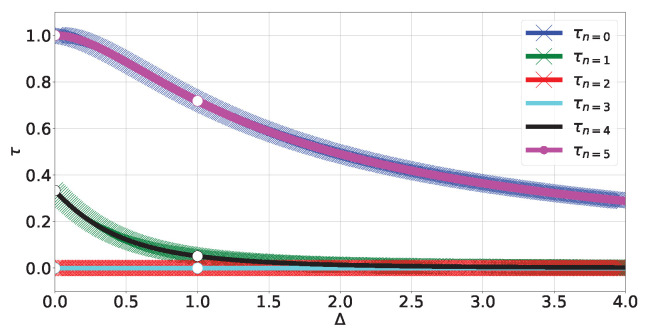
Tangle $H_{XZX}$ levels (A50) and (A52).

**Figure 13:**
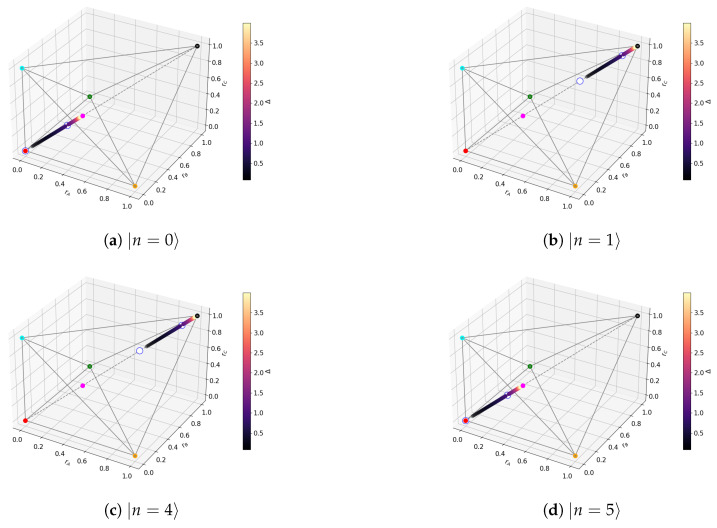
XZX trajectories for Δ∈(0,1)∪(1,4].

Finally, look at the XZX spin chain. This chain’s Hamiltonian contains three-body terms (which are none other than the operators $K_{j}$ defining the cluster state [34] for a closed chain of three qubits) with $X_{j}Z_{j+1}X_{j+2}$ competing against one-body terms $Z_{j}$:

(21) $$\begin{array}{c} H_{\mathrm{XZX}}=-\sum_{j=0}^{2}\left(X_{j}Z_{j+1}X_{j+2}+\Delta Z_{j}\right) \end{array}$$

where Δ≥0, with the energy levels shown in Figure 11 (see (A49) in Appendix C.4). Contrary to the XX and XXX models, the XZX chain presents a tangle that is *robust* (in the sense that the three-body term and one-body term do not commute, so the tangle will not depend on degeneracies) against small perturbations of its Hamiltonian:

(22) $$\begin{array}{c} \tau_{n=0}=\frac{48f_{0}^{3}}{g_{0}^{4}}=\tau_{n=5}=\frac{16f_{5}}{g_{5}^{4}}\;\;\tau_{n=1}=\frac{48f_{0}}{g_{0}^{2}}=\tau_{n=4}=\frac{48f_{4}^{3}}{g_{4}^{4}}\\ \tau_{n=2}=\tau_{n=3}=0 \end{array}$$

where $f_{j},g_{j}$ are functions of Δ defined in (A51). The (Δ,τ) plot is shown in Figure 12. The Bloch-norm representation of the non-degenerate subspaces again forms a trajectory in the polytope restricted to subsets of the main diagonal (see Figure 13), and the shape of the degenerate subspaces is the same as that shown in Figure 6d.

Furthermore, we can see the following emerging patterns:

1. Eigenstates with a *robust* tangle $\in {\vec{V}}_{\mathrm{line}}$. This is because translation invariance causes all three Bloch-norms to be equal. This also explains why the instances of Bloch-norms that are not in the main diagonal correspond to degenerate subspaces: states there generally mix sectors with different momenta. Hence, the states with a robust tangle belong to the GHZ class type 5 subset spanned by simultaneous eigenstates of the translation and parity operators.
2. Out-of-level-crossing degenerate levels in $H_{TFIM}$ and $H_{XZX}$ have a null tangle. This is because, when projected onto those subspaces, the *kinetic* part of the Hamiltonian will commute with the *potential* term $\left[P_{n}H_{0}P_{n},V\right]=0$, causing the tangle to take the same constant value ∀Δ. Since for Δ→∞ the resulting Hamiltonian cannot generate a tangle (since it is a collection of one-qubit operators), then necessarily τ=0.

## 5. Conclusions

In this work, we investigated the geometrical properties of genuine tripartite entanglement in three-qubit states. We derived bounds for the tangle based solely on geometrical insights and arrived at a purely geometrical ansatz for Cayley’s hyperdeterminant of non-generic GHZ states. We then explored how many of these states show up naturally in the energy eigenstate structure of usual spin-chain Hamiltonians. Due to translation and parity symmetries, only a very small subset of GHZ type 5 states will ever appear in states that present a *robust* tangle. Moreover, we identified the necessary conditions for the tangle to be *robust* and not disappear under realistic effects such as symmetry-breaking perturbations and level repulsion.

A possible future direction for this work is the extension of these geometrical arguments to four-qubit systems, where entanglement classification schemes are already known [14,35]. However, this task becomes increasingly difficult as the number of qubits grows due to the calculation of the hyperdeterminant [36]. For three-qubit states, the canonical decomposition circumvented this issue. There are several proposals on how to expand the concept of a canonical decomposition for four-qubit states [37] as well as for general *n*-qubit states [38]; however, it is not yet clear if these simplify the computation in any way [37].

## Figures and Tables

**Table 1:** Values of the local entropies and the tangle for the different classes (from [13]).

Class	$S_{A}$	$S_{B}$	$S_{C}$	τ
A-B-C	0	0	0	0
A-BC	0	>0	>0	0
B-AC	>0	0	>0	0
C-AB	>0	>0	0	0
W	>0	>0	>0	0
GHZ	>0	>0	>0	>0

## Data Availability

The original contributions presented in this study are included in the article. Further inquiries can be directed to the corresponding author.

## Appendix A. Proofs of the Geometrical Properties of the Entanglement Types

We now provide the aforementioned proofs for the polytope structure. We start by proving that all type 3a states lie on the faces of the upper tetrahedron: the CD of a state of such type is $|T_{3a}\rangle =\lambda_{0}|000\rangle +\lambda_{2}|101\rangle +\lambda_{3}|110\rangle$. From it, we compute the Bloch vectors of each qubit by using (5). We obtain that only the *z* components are non-zero. Combining them with the normalization condition yields $z_{A}-z_{B}-z_{C}+1=0$, which is just the scalar equation of a plane. Introducing $s_{I}=\mathrm{sgn}\left(z_{I}\right)$, I∈{A,B,C}, we obtain

(A1) $$\begin{array}{cc} s_{A}r_{A}-s_{B}r_{B}-s_{C}r_{C}+1=0, & \;\mathrm{where}\;s_{\bar{I}}\in \left\{\pm 1\right\}\;\mathrm{and}\;r_{I}\in \left[0,1\right] \end{array}$$

which encodes eight different plane equations (one for each different combination of signs). However, some of them will give a plane that is not present in the ${\left[0,1\right]}^{3}$ cube. These planes are redundant since they can be mapped to the correct ones (the ones present in the cube) via simple translations of the constant +1 in (A1) to −1. The four configurations of signs for each face in the upper tetrahedron $\left(s_{A},s_{B},s_{C}\right)$ are given below.

**Table A1:** Values of the signs for each face of the upper tetrahedron.

$\left(s_{A},s_{B},s_{C}\right)$	Vertices of the Face
(+1,+1,+1)	(0,0,1),(1,1,1),(0,1,0)
(−1,−1,+1)	(0,0,1),(1,1,1),(1,0,0)
(−1,+1,−1)	(1,1,1),(1,0,0),(0,1,0)
(−1,+1,+1)	(0,0,1),(0,1,0),(1,0,0)

This completes the proof. Now, to show that type 2b states lie exclusively in the central diagonal ${\vec{V}}_{\mathrm{line}}$, we just need a direct computation:

(A2) $$z_{A}=z_{B}=z_{C}=\left(2\lambda_{0}^{2}-1\right);\;x_{\bar{I}}=y_{\bar{I}}=0;\Rightarrow r_{A}=r_{B}=r_{C}$$

We now prove that type 3b states span internal triangles of vertices {(0,0,0), (1,1,1), (0,0,1)}, {(0,0,0),(1,1,1),(1,0,0)} and {(0,0,0),(1,1,1),(0,1,0)} for types *1-2*, *2-3* and *1-3*, respectively. We start with a state of type *1-2*, $|T_{3b\text{-}12}\rangle =\lambda_{0}|000\rangle +\lambda_{3}|110\rangle +\lambda_{4}|111\rangle$, and compute the Bloch vectors. From those, we observe that $r_{A}=r_{B}$, so the point $\left(r_{A},r_{B},r_{C}\right)$ must lie on that plane. By using the normalization condition on the λ parameters, one can check that

(A3) $$r_{C}=\sqrt{1-\tau }=\sqrt{r_{A}^{2}+2\lambda_{3}^{2}\left(1+s_{A}r_{A}\right)}>r_{A}$$

The relation in (A3) cannot saturate to equality since that would require $\lambda_{3}=0$, redirecting us back to type 2b. We end up with an allowed region of values $r_{C}>r_{A}=r_{B}$, which is just a triangle of vertices {(1,1,1),(0,0,0),(0,0,1)} (where the edge corresponding to the main diagonal (0,0,0)−(1,1,1) is forbidden). This completes the proof for *1-2*. For *2-3*, $r_{A}$ and $r_{C}$ exchange roles, resulting in a triangle of edges {(1,1,1),(0,0,0),(1,0,0)} from $r_{A}>r_{B}=r_{C}$, and for type *1-3*, $r_{C}$ and $r_{B}$ exchange roles so $r_{B}>r_{A}=r_{C}$, which gives rise to a triangle with vertices {(1,1,1),(0,0,0),(0,1,0)} (in all of these the central diagonal (0,0,0)−(1,1,1) is forbidden). In summary,

(A4) $$\begin{array}{cccccc} |T_{3b\text{-}12}\rangle \to & r_{C}>r_{A}=r_{B}; & |T_{3b\text{-}23}\rangle \to & r_{A}>r_{C}=r_{B}; & |T_{3b\text{-}13}\rangle \to & r_{B}>r_{A}=r_{C}. \end{array}$$

Finally, we show now where type 4b states lie. We start with $\lambda_{2}=0$: $|T_{4b,2}\rangle =\lambda_{0}|000\rangle +\lambda_{1}e^{i\varphi }|110\rangle +\lambda_{3}|110\rangle +\lambda_{4}|111\rangle$ and notice that it is just a normalized sum of a $|T_{3b\text{-}12}\rangle$ state and a $|T_{3b\text{-}23}\rangle$ state, which one would naively expect to lie somewhere between both those states. To put this into more solid terms, we compute the Bloch-norms

(A5) $$\begin{array}{cccc} r_{A}^{2}=\left(1-\tau \right)-4\lambda_{0}^{2}\lambda_{3}^{2}; & \;r_{B}^{2}=r_{A}^{2}-4\lambda_{1}^{2}\lambda_{4}^{2}; & \;r_{C}^{2} & =\left(1-\tau \right)-4\lambda_{1}^{2}\lambda_{4}^{2} \end{array}$$

where we have substituted $4\lambda_{2}^{2}\lambda_{4}^{2}$ with τ as per the *tangle master equation* (10). From these, we can obtain

(A6) $$\begin{array}{c} r_{B}^{2}<r_{A}^{2}<\left(1-\tau \right)\to r_{B}<r_{A}<\sqrt{1-\tau }\;\\ r_{B}^{2}<r_{C}^{2}<\left(1-\tau \right)\to r_{B}<r_{C}<\sqrt{1-\tau } \end{array}$$

where in the → step we have used that the square root is monotonous in the [0,1] interval. Comparing this with the cases *1-2* and *2-3* in (A4), we see that indeed this implies that the $|T_{4b,2}\rangle$ states reside in the space in between those two planes (with the planes themselves being forbidden). The same procedure can be repeated for the case in which $\lambda_{3}=0$ instead, and one finds that the allowed zone is in between the planes of types *2-3* and *1-3*. This completes the proof.

## Appendix B. (*R*, $\vec{\lambda }$) Surface Fibration by λ → Curves

We illustrate for this one the general method one can follow to reproduce our results for the bounds presented in Section 3. We start by looking at the case $\vec{\lambda }=\left(0,0,\lambda_{3}\right)$. Substituting this into (9) and then using the normalization condition to remove $\lambda_{4}$ gives

(A7) $$R^{2}=12\lambda_{0}^{4}+4\lambda_{0}^{2}\lambda_{3}^{2}-12\lambda_{0}^{2}+3\to {\left(\lambda_{0}^{2}\right)}_{\pm }=\frac{1}{2}-\frac{\lambda_{3}^{2}}{6}\pm \frac{\sqrt{3R^{2}+\lambda_{3}^{4}-6\lambda_{3}^{2}}}{6}$$

Substituting ${\left(\lambda_{0}^{2}\right)}_{\pm }$ into Equation (10) will yield two solutions. The correct one,

(A8) $$\tau \left(R,\lambda_{3}\right)=\tau_{M}\left(R\right)+\frac{4\lambda_{3}^{2}}{9}\cdot \left(\lambda_{3}^{2}-3-\sqrt{3R^{2}+\lambda_{3}^{2}\left(\lambda_{3}^{2}-6\right)}\right),$$

is the one that satisfies the consistency condition:

(A9) $$\min_{R}\left\{\underset{\left(R,\lambda_{3}\right)}{\mathrm{argmin}}\left[\tau \left(R,\lambda_{3}\right)\right]\right\}=1$$

Our next step will be to attempt to *fibrate* the surface (A8) with curves $\lambda_{3}=\lambda_{3}\left(R\right)$ and then find the one curve that allows us to minimize the value of τ for a given value of *R*. Imposing now the reality condition τ∈R yields the following inequality:

(A10) $$3R^{2}+\lambda_{3}^{4}-6\lambda_{3}^{2}\geq 0,\;\mathrm{which}\;\mathrm{saturates}\;\mathrm{for}\;{\left(\lambda_{3,\mathrm{sat}}^{2}\left(R\right)\right)}_{\pm }=3\pm \sqrt{9-3R^{2}}$$

We can now use a fact we learned from the analysis of the Bloch-norm diagram from the previous section: *as R→0, the three-qubit state $\to |\mathrm{GHZ}\rangle$*. Imposing this constraint is equivalent to imposing $\lim_{R\to 0}\lambda_{3}\left(R\right)=0$, and it reveals that the correct solution is necessarily the (−) one. With this, the τ-reality condition is also telling us that $\lambda_{3}\leq \sqrt{{\left(\lambda_{3,\mathrm{sat}}^{2}\right)}_{-}}$. By substituting the correct solution back into (A8), we arrive at

(A11) $$\begin{array}{c} \tau \left(R,\lambda_{3}=\sqrt{{\left(\lambda_{3,\mathrm{sat}}^{2}\left(R\right)\right)}_{-}}\right)=5\tau_{M}\left(R\right)-4\sqrt{\tau_{M}\left(R\right)} \end{array}$$

There exist values of *R* for which ${\left(\lambda_{3,\mathrm{sat}}^{2}\left(R\right)\right)}_{-}$ will be greater than 1, which is forbidden since by construction $0<\lambda_{3}<1$. What this is telling us is that the solution has only a certain range of values of *R* for which it is valid. This range is $R\leq R_{o}:=\sqrt{5/3}<\sqrt{3}$. Moreover, it does not fit the requirement observed in Figure 3b,c to make the tangle vanish at R=1, meaning that this is not the curve we are looking for. Instead, we want a curve $\lambda_{3}^{\left(★\right)}\left(R\right)$ such that $\tau_{-}\left(R,\lambda_{3}^{\left(★\right)}\left(R\right)\right)=\tau_{★}\left(R\right)$, which must fulfill the requirement $\tau_{-}\left(R=1,\lambda_{3}^{\left(★\right)}\left(R=1\right)\right)=0$. We can solve analytically for this, finding $\lambda_{3}^{\left(★\right)}\left(R=1\right)=1/\sqrt{2}$. Moreover, in the small *R* limit, the curve that maximizes $\lambda_{3}$ will be the one minimizing τ, so $\lambda_{3}^{\left(★\right)}\left(R\right)$ must fulfill the following condition:

(A12) $$\begin{array}{cc} \lim_{R\to 0}\lambda_{3}^{\left(★\right)}\left(R\right)≃\sqrt{{\left(\lambda_{3,\mathrm{sat}}^{2}\left(R\right)\right)}_{-}}=\sqrt{3-\sqrt{9-3R^{2}}} & \;\;\lim_{R\to 1}\lambda_{3}^{\left(★\right)}\left(R\right)≃\lambda_{3}^{\left(a\right)}\left(R\right)=\frac{R}{\sqrt{2}} \end{array}$$

One can check numerically that the change from one curve to the other becomes appreciable around R≃0.56. So, we finally arive at our approximated solution for the $\lambda_{3}^{\left(★\right)}\left(R\right)$ curve such that $\tau \left(R,\lambda_{3}=\lambda_{3}^{\left(★\right)}\left(R\right)\right)=\tau_{★}\left(R\right)$:

(A13) $$\lambda_{3}^{\left(★\right)}\left(R\right)≃\left\{\begin{array}{cc} \sqrt{3-\sqrt{9-3R^{2}}} & \;\mathrm{if}\;R\overset{<}{∼}0.56\\ R/\sqrt{2} & \;\mathrm{otherwise}\; \end{array}\right\}$$

which when substituted back into (A8) will yield (12).

For the case of type 4c states, the same procedure can be employed. The only difference being that in this case we have two curves to worry about, one for $\lambda_{2}$ and another for $\lambda_{3}$:

(A14) $$\begin{array}{c} \tau \left(R,\lambda_{2},\lambda_{3}\right)=\tau_{M}\left(R\right)-\frac{16\lambda_{2}^{2}\lambda_{3}^{2}}{9}+\frac{4\left(\lambda_{2}^{4}+\lambda_{3}^{4}\right)}{9}\\ +\frac{4\left(\lambda_{2}^{2}+\lambda_{3}^{2}\right)}{9}\left(-3+\sqrt{3R^{2}+\left(\lambda_{2}^{4}+\lambda_{3}^{4}\right)-6\left(\lambda_{2}^{2}+\lambda_{3}^{2}\right)+26\lambda_{2}^{2}\lambda_{3}^{2}}\right) \end{array}$$

which makes the computations more cumbersome. The end results are (13).

Finally, we now show where the (A19) and (A20) results come from: we start by considering an arbitrary point $\vec{r}\in {\left[0,1\right]}^{3}$, and then the distance from that point to the straight line spanned by the main diagonal is the length of the vector connecting it to a point on the line such that this vector is perpendicular to the line. A simple trigonometric calculation gives

(A15) $$d\left(\vec{r},{\vec{V}}_{\mathrm{line}}\right)=\sqrt{\frac{2}{3}}\sqrt{R^{2}-\sum_{i<j}\left(r_{i}r_{j}\right)}$$

Take a state $|T_{3b\text{-}12}\rangle$ (such as the one we used for the type 3b geometry proof in Appendix A); then the distance to the main diagonal of the lowest order is

(A16) $$\begin{array}{cc} {\left[d\left(\vec{r}\left(|T_{3b\text{-}12}\rangle \right),{\vec{V}}_{\mathrm{line}}\right)\right]}^{2} & ≃\frac{8\lambda_{0}^{4}\lambda_{3}^{4}}{3r_{C}^{2}}\left(1+\frac{2\lambda_{0}^{2}\lambda_{3}^{2}}{r_{C}^{2}}+O\left({\left[\frac{\lambda_{0}^{2}\lambda_{3}^{2}}{r_{C}^{2}}\right]}^{4}\right)\right) \end{array}$$

so at the lowest order

(A17) $$-\lambda_{3}^{2}\left(\lambda_{3}^{2}-3-\sqrt{3R^{3}+\lambda_{3}^{2}\left(\lambda_{3}^{2}-6\right)}\right)≃-3r_{C}\sqrt{\frac{3}{2}}d\left(\vec{r}\left(|T_{3b\text{-}12}\rangle \right),{\vec{V}}_{\mathrm{line}}\right)$$

so by direct comparison to (A8), one obtains (14), where

(A18) $$F\left(\vec{r}\left(|T_{3b\text{-}12}\rangle \right)\right)≃2r_{C}\sqrt{\frac{2}{3}}+O\left(r_{C}^{2}\right)$$

For the other type 3b states, we can use the fact that the only difference in geometrical behavior is $r_{C}↔r_{A}$ and $r_{C}↔r_{B}$ for $|T_{3b\text{-}23}\rangle$ and $|T_{3b\text{-}13}\rangle$, respectively (see (A4)), to find

(A19) $$\begin{array}{cc} F\left(\vec{r}\left(|T_{3b\text{-}23}\rangle \right)\right)≃2r_{A}\sqrt{\frac{2}{3}}+O\left(r_{A}^{2}\right)\; & \;F\left(\vec{r}\left(|T_{3b\text{-}13}\rangle \right)\right)≃2r_{B}\sqrt{\frac{2}{3}}+O\left(r_{B}^{2}\right) \end{array}$$

which tells us that F somehow encodes the geometrical asymmetries of the Bloch-norm representations of the states.

Furthermore, we can follow the same procedure for type 4b states as well:

(A20) $$\begin{array}{cc} F\left(\vec{r}\left(|T_{4b\text{-}1}\rangle \right)\right)≃2\sqrt{3}r_{B}+O\left(r_{B}^{2}\right)\; & \;F\left(\vec{r}\left(|T_{4b\text{-}2}\rangle \right)\right)≃2\sqrt{3}r_{C}+O\left(r_{C}^{2}\right) \end{array}$$

## Appendix C. Analytic Details of Three-Qubit Spin Chains

It is possible to code a function that performs the CD procedure analytically for a simple enough input three-qubit state. We have done so for the eigenstates of the chain Hamiltonians considered in Section 4 and we show the results here.

Such results depend, in general, on which one of the two possible solutions to the $\det\left(T_{0}^{\prime }\right)=0$ equation of the CD procedure is chosen:

(A21) $$\begin{array}{cc} U=\left(\begin{array}{cc} z & w\\ -w^{*} & z^{*} \end{array}\right)|{\left|z\right|}^{2}+{\left|w\right|}^{2}=1 & \;\mathrm{and}\;\det\left(T_{0}^{\prime }\right)=0,\;\mathrm{where}\;T_{i}^{\prime }=\sum_{j}U_{ij}T_{j} \end{array}$$

which means one must specify which solution is picked each time. The exception to this is the tangle, which is the same for both solutions [15].

We will label the energy levels by their integer ordering *n* (with n=0 corresponding to the ground state) and the degeneracies by the integer $m_{n}=\dim\left(\mathrm{span}(\mathrm{subspace}\;n)\right)$. We will also label the eigenstates by their eigenvalues under conserved quantities such as parity *p* and momentum number *k*. For the XX and XXX models, the magnetization *m* is conserved, so it will be used as well.

### Appendix C.1. TFIM Analytics

Let us first consider the TFIM (16). It can be solved exactly, giving an energy spectrum:

(A22) $$\begin{array}{ccc} E_{0} & =-\Delta -2\sqrt{\Delta^{2}-\Delta +1}-1; & \;m=1\\ E_{1} & =\Delta -2\sqrt{\Delta^{2}+\Delta +1}-1; & \;m=1\\ E_{2} & =-\Delta +2\sqrt{\Delta^{2}-\Delta +1}-1; & \;m=1\\ E_{3} & =1-\Delta ; & \;m=2\\ E_{4} & =\Delta +1; & \;m=2\\ E_{5} & =\Delta +2\sqrt{\Delta^{2}+\Delta +1}-1; & \;m=1 \end{array}$$

where the degeneracy count remains valid outside of any level-crossing point (like Δ=0, which corresponds to a quantum critical point, or Δ=1). At any level-crossing point, the energy expressions are still valid but the degeneracy count will change, so calculations need to be performed again at these points for the relevant levels that are fusing together.

#### Appendix C.1.1. TFIM Outside Level-Crossing Points

We start by computing the energy eigenstates outside of these two level-crossing points:

(A23) $$\begin{array}{cc} |n=0,p=+1,k=0\rangle = & \frac{1}{g_{0}}\left[f_{0}|000\rangle +\sqrt{3}{\hat{X}}^{⊗3}|W\rangle \right]\\ |n=1,p=-1,k=0\rangle = & \frac{f_{1}}{g_{1}}\sqrt{3}|W\rangle +\frac{3}{g_{1}}{\hat{X}}^{⊗3}|000\rangle \\ |n=2,p=+1,k=0\rangle = & \frac{1}{g_{2}}\left[-f_{2}|000\rangle +\sqrt{3}{\hat{X}}^{⊗3}|W\rangle \right]\\ |n=5,p=-1,k=0\rangle = & \frac{-f_{5}}{g_{5}}\sqrt{3}|W\rangle +\frac{3}{g_{5}}{\hat{X}}^{⊗3}|000\rangle \end{array}$$

for which we can calculate analytically the Bloch-norm vectors as well:

(A24) $$\begin{array}{cc} |n=0,2\rangle \to & \left(r_{A},r_{B},r_{C}\right)=\left(\frac{\left|f_{n}^{2}-1\right|}{g_{n}^{2}}\right)\cdot \left(1,1,1\right)\\ |n=1,5\rangle \to & \left(r_{A},r_{B},r_{C}\right)=\left(\frac{\left|f_{n}^{2}-9\right|}{g_{n}^{2}}\right)\cdot \left(1,1,1\right) \end{array}$$

For the degenerate states,

(A25) $$\begin{array}{cc} |n=3,p=-1,k=1\rangle = & |W_{k=1}\rangle \\ |n=3,p=-1,k=2\rangle = & |W_{k=2}\rangle \\ |n=3,p=-1,(\alpha ,\beta )\rangle = & \alpha |n=3,p=-1,k=1\rangle +\beta |n=3,p=-1,k=2\rangle ;\\ |n=4,p=+1,k=1\rangle = & {\hat{X}}^{⊗3}|W_{k=1}\rangle \\ |n=4,p=+1,k=2\rangle = & {\hat{X}}^{⊗3}|W_{k=2}\rangle \\ |n=4,p=-1,(\alpha ,\beta )\rangle = & \alpha |n=4,p=+1,k=1\rangle +\beta |n=4,p=+1,k=2\rangle ; \end{array}$$

(A26) $$\begin{array}{ccc} |n=3,(\alpha ,\beta )\rangle & \to \left(r_{A},r_{B},r_{C}\right)=\frac{1}{3}( & \left|1+2|\alpha |\beta \left[\cos\arg(\alpha )+\sqrt{3}\sin\arg(\alpha )\right]\right|,\\ & & \left|1+2|\alpha |\beta \left[\cos\arg(\alpha )-\sqrt{3}\sin\arg(\alpha )\right]\right|,\\ & & \left|1-4|\alpha |\beta \cos\arg(\alpha )\right|)\\ |n=4,(\alpha ,\beta )\rangle & \to \;\mathrm{same}\;\mathrm{as}\;\mathrm{for}\;n\;=\;3 & \end{array}$$

where α∈C and $\beta \in R^{+}$ such that ${\left|\alpha \right|}^{2}+\beta^{2}=1$ and we have already used the global U(1) to remove the complex phase. Also, the states $|W_{k=1}\rangle$ and $|W_{k=2}\rangle$ are defined as

(A27) $$\begin{array}{c} |W_{k=1}\rangle :=\frac{1}{\sqrt{3}}\left(|001\rangle +e^{\left(i\frac{2\pi }{3}\right)}|010\rangle +e^{\left(i\frac{4\pi }{3}\right)}|100\rangle \right)\\ |W_{k=2}\rangle :=\frac{1}{\sqrt{3}}\left(|001\rangle +e^{\left(i\frac{4\pi }{3}\right)}|010\rangle +e^{\left(i\frac{2\pi }{3}\right)}|100\rangle \right) \end{array}$$

The degeneracy allows for arbitrary superpositions characterized by the parameters α and β.

For the non-degenerate subspaces, the $f_{j},g_{j}$ parameters on (A23) are defined as

(A28) $$\begin{array}{cccc} f_{0} & =-1+2\Delta +2b(\Delta )\geq 0; & g_{0} & =\sqrt{f_{0}^{2}+3}>0\\ f_{1} & =2\Delta +2a(\Delta )+1\geq 0; & g_{1} & =\sqrt{3}\sqrt{f_{1}^{2}+3}>0\\ f_{2} & =\left[-2\Delta +1+2b(\Delta )\right]\geq 0; & g_{2} & =\sqrt{f_{2}^{2}+3}>0\\ f_{5} & =\left[-2\Delta -1+2a(\Delta )\right]\geq 0; & g_{5} & =\sqrt{3}\sqrt{f_{5}^{2}+3}>0 \end{array}$$

(A29) $$\begin{array}{cccc} b(\Delta ) & =\sqrt{1-\Delta +\Delta^{2}}; & a(\Delta ) & =\sqrt{1+\Delta +\Delta^{2}}; \end{array}$$

Now that we have the exact eigenstates at hand, we can begin computing the CD and the tangle for each level. For n=0, we have that the two solutions of Equation (A21) are

(A30) $$w=\pm \frac{\sqrt{f_{0}}}{\sqrt{f_{0}+1}}\;\;z=\frac{1}{\sqrt{f_{0}+1}}$$

of which we pick the + solution. The resulting λ parameters are

(A31) $$\begin{array}{ccccc} \lambda_{0}=\frac{\sqrt{f_{0}+1}}{g_{0}}; & \lambda_{1}=\frac{\sqrt{f_{0}}\left|f_{0}-1\right|}{g_{0}\sqrt{f_{0}+1}}; & \lambda_{2}=\lambda_{3}=\frac{\left|f_{0}-1\right|}{g_{0}\sqrt{f_{0}+1}}; & \lambda_{4}=\frac{2\sqrt{f_{0}}}{g_{0}\sqrt{f_{0}+1}}; & \varphi =0 \end{array}$$

For the first excited state $|n=1\rangle$, the solutions to (A21) are

(A32) $$w=\frac{\pm \sqrt{f_{1}}}{\sqrt{f_{1}+3}}\;\;z=\frac{\sqrt{3}}{\sqrt{f_{1}+3}}$$

of which we pick the + solution. The resulting λ parameters are

(A33) $$\begin{array}{cc} \lambda_{0}=\frac{\sqrt{f_{1}}\sqrt{f_{1}+3}}{g_{1}}; & \lambda_{1}=\frac{\sqrt{3}\left|f_{1}-3\right|}{g_{1}\sqrt{f_{1}+3}};\;\lambda_{2}=\lambda_{3}=\frac{\sqrt{f_{1}}\left|f_{1}-3\right|}{g_{1}\sqrt{f_{1}+3}};\;\lambda_{4}=\frac{2\sqrt{3}f_{1}}{g_{1}\sqrt{f_{1}+3}};\;\varphi =0 \end{array}$$

For the second excited state $|n=2\rangle$, the solutions to (A21) are

(A34) $$w=\frac{\pm i\sqrt{f_{2}}}{\sqrt{f_{2}+1}}\;\;z=\frac{1}{\sqrt{f_{2}+1}}$$

of which we pick the + solution. The resulting λ parameters are

(A35) $$\begin{array}{ccccc} \lambda_{0}=\frac{\sqrt{f_{2}+1}}{g_{2}}; & \;\lambda_{1}=\frac{\sqrt{f_{2}}\left|f_{2}-1\right|}{g_{2}\sqrt{f_{2}+1}}; & \;\lambda_{2}=\lambda_{3}=\frac{\left|f_{2}-1\right|}{g_{2}\sqrt{f_{2}+1}}; & \;\lambda_{4}=\frac{2\sqrt{f_{2}}}{g_{2}\sqrt{f_{2}+1}}; & \;\varphi =0 \end{array}$$

For the third excited subspace, we compute the CD for the arbitrary superposition $|n=3;\left(\alpha ,\beta \right)\rangle$:

(A36) $$\lambda_{0}=\frac{\left|\alpha e^{-i\frac{2\pi }{3}}+\beta e^{+i\frac{2\pi }{3}}\right|}{\sqrt{3}},\;\lambda_{1}=0=\lambda_{4},\;\lambda_{2}=\left|\frac{\alpha +\beta }{\sqrt{3}}\right|,\;\lambda_{3}=\left|\frac{\alpha e^{-i\frac{2\pi }{3}}+\beta }{\sqrt{3}}\right|$$

For the fourth excited subspace, since $|4,(\alpha ,\beta )\rangle ={\hat{X}}^{⊗3}|3,(\alpha ,\beta )\rangle$, the CD is the same as for n=3. For the fifth excited state $|n=5\rangle$, the solutions to (A21) are

(A37) $$w=\frac{\pm i\sqrt{f_{5}}}{\sqrt{f_{5}+3}}\;\;z=\frac{\sqrt{3}}{\sqrt{f_{5}+3}}$$

of which we pick the + solution. The resulting λ parameters are

(A38) $$\begin{array}{ccccc} \lambda_{0}=\frac{\sqrt{f_{5}}\sqrt{f_{5}+3}}{g_{5}}; & \;\lambda_{1}=\lambda_{3}=\frac{\sqrt{3}\left|f_{5}-3\right|}{g_{5}\sqrt{f_{5}+3}}; & \;\lambda_{2}=\frac{\sqrt{f_{5}}\left|f_{5}^{2}-9\right|}{g_{5}{\left(f_{5}+3\right)}^{\frac{3}{2}}}; & \;\lambda_{4}=\frac{2\sqrt{3}f_{5}}{g_{5}\sqrt{f_{5}+3}}; & \;\varphi =0 \end{array}$$

#### Appendix C.1.2. TFIM at Level-Crossing Points

Consider now the level-crossing point Δ=1. Almost all levels are unchanged here, so the results for Δ outside the level crossing still apply, the exception being levels n=2 and n=3, which fuse at this point. By diagonalizing the Hamiltonian at this value of Δ, it is possible to obtain a new basis for the degenerate subspace:

(A39) $$\begin{array}{cc} |n=2,s=1\rangle & =\frac{1}{2}\left[-|000\rangle +|011\rangle +|101\rangle +|110\rangle \right]\\ |n=2,s=1\rangle & =|W_{k=1}\rangle \\ |n=2,s=3\rangle & =|W_{k=2}\rangle \\ |n=2,\left(\alpha ,\ldots \right)\rangle & =\gamma |2,1\rangle +\alpha |2,2\rangle +\beta |2,3\rangle , \end{array}$$

with $\alpha ,\gamma \in C\;\mathrm{and}\;\beta \in R^{+}$, which are the coefficients of an arbitrary superposition in this subspace. The hyperdeterminant formula allows us to directly obtain the tangle:

(A40) $$\tau_{n=2,\Delta =1}={\left|\gamma \right|}^{2}\left|\gamma^{2}-\frac{1}{3}{\left[(\alpha +\beta )-(\xi -\eta )\right]}^{2}+\frac{4\eta }{3}\left(\alpha +\beta \right)\right|$$

where $\xi =\left(\alpha e^{-i\frac{2\pi }{3}}+\beta e^{+i\frac{2\pi }{3}}\right)$ and $\eta =\left(\alpha e^{+i\frac{2\pi }{3}}+\beta e^{-i\frac{2\pi }{3}}\right)$. We can see immediately that γ=0⇒τ=0, meaning that the entire tangle in the superposition comes solely from the $|n=2,s=1\rangle$ state in the subspace.

### Appendix C.2. XX Analytics

We now consider the XX chain (18). The energy spectrum is

(A41) $$\begin{array}{ccc} E_{0} & =-\Delta -4; & \;m=1\\ E_{1} & =+\Delta -4; & \;m=1\\ E_{2} & =-3\Delta ; & \;m=1\\ E_{3} & =+3\Delta ; & \;m=1\\ E_{4} & =2-\Delta ; & \;m=2\\ E_{5} & =2+\Delta ; & \;m=2 \end{array}$$

#### XX Outside Level-Crossing Points

We start by computing the energy eigenstates outside of level-crossing points:

(A42) $$\begin{array}{cc} |n=0,k=0,m=+1,p=-1\rangle & =|W\rangle \\ |n=1,k=0,m=-1,p=+1\rangle & ={\hat{X}}^{⊗3}|W\rangle \\ |n=2,k=0,m=+3,p=+1\rangle & =|000\rangle \\ |n=3,k=0,m=-3,p=-1\rangle & =|111\rangle \\ |n=4,k=1,m=+1,p=-1\rangle & =|W_{k=1}\rangle \\ |n=4,k=2,m=+1,p=-1\rangle & =|W_{k=2}\rangle \\ |n=4;m=+1,p=-1,(\alpha ,\beta )\rangle & =\beta |4,k=1\rangle +\alpha |4,k=2\rangle ;\\ |n=5,k=1,m=-1,p=+1\rangle & ={\hat{X}}^{⊗3}|W_{k=1}\rangle \\ |n=5,k=2,m=-1,p=+1\rangle & ={\hat{X}}^{⊗3}|W_{k=2}\rangle \\ |n=5;m=-1,p=+1,(\alpha ,\beta )\rangle & =\beta |5,k=1\rangle +\alpha |5,k=2\rangle , \end{array}$$

where α and β are defined as in (A25). We can calculate analytically the Bloch-norms for the non-degenerate levels:

(A43) $$|n=0\;\&1\rangle \to \frac{1}{3}\left(1,1,1\right)\;\;|n=2\;\&3\rangle \to \left(1,1,1\right)$$

We can now compute the CD for each level: For n=0, since it is plainly the W state we have

(A44) $$\lambda_{0}=\lambda_{2}=\lambda_{3}=\frac{1}{\sqrt{3}};\;\lambda_{1}=\lambda_{4}=0;$$

For the n=1 subspace, the result of the CD is the same. For n=3 and n=4, one has $\lambda_{0}=1$ and $\lambda_{n>0}=0$ since they are product states. For both n=4 and n=5, one has that $\lambda_{1}=\lambda_{4}=0$, while the non-zero λ are the same as we saw for levels n=3 and 4 for the TFIM (A36).

### Appendix C.3. XXX Analytics

We now consider the XX chain (19). The energy spectrum is

(A45) $$\begin{array}{ccc} E_{0} & =+3\Delta ; & \;m=2\\ E_{1} & =4-\Delta ; & \;m=2\\ E_{2} & =-\Delta -2; & \;m=4 \end{array}$$

for Δ∈R. At Δ=−1/2, there is a level crossing, where the role of the GS changes from n=0 to n=1. We will still keep the labels used in Δ∈(−∞,−1/2) for consistency.

#### XXX Outside Level-Crossing Points

We start by computing the energy eigenstates outside of level-crossing points:

(A46) $$\begin{array}{cc} |n=0,k=0,m=+3,p=+1\rangle & =|000\rangle \\ |n=0,k=0,m=-3,p=-1\rangle & =|111\rangle \\ |n=0,k=0,\left(\alpha ,\beta \right)\rangle & =\beta |000\rangle +\alpha |111\rangle \\ |n=1,k=0,m=-1,p=+1\rangle & ={\hat{X}}^{⊗3}|W\rangle \\ |n=1,k=0,m=+1,p=-1\rangle & =|W\rangle \\ |n=1,k=0,m=+1,\;\left(\alpha ,\beta \right)\rangle & =\beta {\hat{X}}^{⊗3}|W\rangle +\alpha |W\rangle \\ |n=2,k=1,m=+1,p=-1\rangle & =|W_{k=1}\rangle \\ |n=2,k=1,m=-1,p=+1\rangle & ={\hat{X}}^{⊗3}|W_{k=1}\rangle \\ |n=2,k=2,m=+1,p=-1\rangle & =|W_{k=2}\rangle \\ |n=2,k=2,m=-1,p=+1\rangle & ={\hat{X}}^{⊗3}|W_{k=2}\rangle \\ |n=2,\left(\alpha ,\ldots \delta \right)\rangle & =\alpha |2,1\rangle +\beta |2,2\rangle +\gamma |2,3\rangle +\delta |2,4\rangle \end{array}$$

We can compute the CD for the n=0 subspace, obtaining

(A47) $$\lambda_{0}=\beta ;\;\lambda_{4}=\left|\alpha \right|;\;\lambda_{j}=0\;\mathrm{for}\;j=1,2,3;$$

For n=1, the CD is

(A48) $$\begin{array}{ccc} \lambda_{0}=\frac{1}{\sqrt{3}}; & \lambda_{2}=\lambda_{3}=\sqrt{\frac{1}{3}+\beta^{2}\left(\beta^{2}-1\right)}; & \lambda_{4}=\beta \sqrt{1-\beta^{2}};\\ & \varphi =\frac{\pi }{6}-2\arg\left(\alpha \right)+\mathrm{atan}\left(\frac{-\beta^{2}\sqrt{3}}{2-3\beta^{2}}\right); \end{array}$$

but for level n=2, the degeneracy is so large that computing the CD is not possible due to a timeout error from the automated solver, but one can still obtain the tangle from the hyperdeterminant formula (20).

### Appendix C.4. XZX Analytics

The energy spectrum of the XZX chain (21) reads

(A49) $$\begin{array}{ccc} E_{0} & =+\Delta -2a(\Delta )-1; & \;m=1\\ E_{1} & =-\Delta -2a(\Delta )+1; & \;m=1\\ E_{2} & =-1+\Delta ; & \;m=2\\ E_{3} & =+1-\Delta ; & \;m=2\\ E_{4} & =+\Delta +2a(\Delta )-1; & \;m=1\\ E_{5} & =-\Delta +2a(\Delta )+1; & \;m=1 \end{array}$$

where a(Δ) is defined as in (A29).

#### XZX Outside Level-Crossing Points

The energy eigenstates outside of level-crossing points are

(A50) $$\begin{array}{cc} |n=0,p=-1,k=0\rangle & =\frac{1}{g_{0}}\left[3|111\rangle +-f_{0}\sqrt{3}|W\rangle \right]\\ |n=1,p=+1,k=0\rangle & =\frac{1}{g_{0}}\left[\sqrt{3}f_{0}|000\rangle +3{\hat{X}}^{⊗3}|W\rangle \right]\\ |n=4,p=-1,k=0\rangle & =\frac{1}{g_{4}}\left[3|111\rangle +\sqrt{3}f_{4}|W\rangle \right]\\ |n=5,p=+1,k=0\rangle & =\frac{1}{g_{5}}\left[-f_{5}|000\rangle +\sqrt{3}{\hat{X}}^{⊗3}|W\rangle \right] \end{array}$$

for the non-degenerate levels, with the $f_{j},g_{j}$ defined as

(A51) $$\begin{array}{cccc} f_{0} & =2\Delta +2a(\Delta )+1\geq 0; & g_{0} & =\sqrt{3}\sqrt{f_{0}^{2}+3}\\ f_{4} & =-2\Delta +2a-1\geq 0 & g_{4} & =\sqrt{3}\sqrt{f_{4}^{2}+3}\\ f_{5} & =f_{4}\geq 0 & g_{5} & =\sqrt{f_{5}^{2}+3} \end{array}$$

while for the degenerate levels,

(A52) $$\begin{array}{cc} |n=2,p=+1,k=1\rangle & ={\hat{X}}^{⊗3}|W_{k=1}\rangle \\ |n=2,p=+1,k=2\rangle & ={\hat{X}}^{⊗3}|W_{k=2}\rangle \\ |n=2,p=+1,(\alpha ,\beta )\rangle & =\alpha |2,1\rangle +\beta |2,2\rangle ;\\ |n=3,p=-1,k=1\rangle & =|W_{k=1}\rangle \\ |n=3,p=-1,k=2\rangle & =|W_{k=2}\rangle \\ |n=3,p=-1,(\alpha ,\beta )\rangle & =\beta |3,1\rangle +\alpha |3,2\rangle ;. \end{array}$$

We can now compute the CD of the eigenstates. Starting with n=0, the solutions to (A21) are

(A53) $$w=\frac{\pm i\sqrt{f_{0}}}{\sqrt{f_{0}+3}};\;z=\frac{\sqrt{3}}{\sqrt{f_{0}+3}}$$

and picking the (+) solution,

(A54) $$\begin{array}{ccccc} \lambda_{0}=\frac{\sqrt{f_{0}}\sqrt{f_{0}+3}}{g_{0}}; & \;\lambda_{1}=\frac{\sqrt{3}\left|f_{0}-3\right|}{g_{0}\sqrt{f_{0}+3}}; & \;\lambda_{2}=\lambda_{3}=\frac{\sqrt{f_{0}}\left|f_{0}-3\right|}{g_{0}\sqrt{f_{0}+3}}; & \;\lambda_{4}=\frac{2\sqrt{3}f_{0}}{g_{0}\sqrt{f_{0}+3}}; & \;\varphi =0 \end{array}$$

For n=1, the solutions are

(A55) $$w=\frac{\pm \sqrt{f_{0}}}{\sqrt{f_{0}+1}};\;z=\frac{1}{\sqrt{f_{0}+1}}$$

and we pick the (+) one:

(A56) $$\begin{array}{c} \begin{array}{ccc} \lambda_{0}=\frac{\sqrt{f_{0}+1}}{\sqrt{f_{0}^{2}+3}}; & \;\lambda_{1}=\frac{\sqrt{f_{0}}\left|f_{0}-1\right|}{\sqrt{f_{0}+1}\sqrt{f_{0}^{2}+3}}; & \;\lambda_{2}=\lambda_{3}=\frac{\left|f_{0}-1\right|}{\sqrt{f_{0}+1}\sqrt{f_{0}^{2}+3}}; \end{array}\\ \lambda_{4}=\frac{2\sqrt{f_{0}}}{\sqrt{f_{0}+1}\sqrt{f_{0}^{2}+3}};\;\;\varphi =0 \end{array}$$

For n=2 and n=3, we can reuse the results from levels n=3 and 4 of the TFIM. For n=4, the solutions are

(A57) $$w=\frac{\pm \sqrt{f_{4}}}{\sqrt{f_{4}+3}};\;z=\frac{\sqrt{3}}{\sqrt{f_{4}+3}}$$

and we pick the (+) one:

(A58) $$\begin{array}{ccccc} \lambda_{0}=\frac{\sqrt{f_{4}}\sqrt{f_{4}+3}}{g_{4}}; & \;\lambda_{1}=\frac{\sqrt{3}\left|f_{4}-3\right|}{g_{4}\sqrt{f_{4}+3}}; & \;\lambda_{2}=\lambda_{3}=\frac{\sqrt{f_{4}}\left|f_{4}-3\right|}{g_{4}\sqrt{f_{4}+3}}; & \;\lambda_{4}=\frac{2\sqrt{3}f_{4}}{g_{4}\sqrt{f_{4}+3}}; & \;\varphi =0 \end{array}$$

Finally, for n=5,

(A59) $$w=\frac{\pm i\sqrt{f_{5}}}{\sqrt{f_{5}+1}};\;z=\frac{1}{\sqrt{f_{5}+1}}$$

(A60) $$\begin{array}{ccccc} \lambda_{0}=\frac{\sqrt{f_{5}+1}}{g_{5}}; & \;\lambda_{1}=\frac{\sqrt{f_{5}}\left|f_{5}-1\right|}{g_{5}\sqrt{f_{5}+1}}; & \;\lambda_{2}=\lambda_{3}=\frac{\left|f_{5}-1\right|}{g_{5}\sqrt{f_{5}+1}}; & \;\lambda_{4}=\frac{2\sqrt{f_{5}}}{g_{5}\sqrt{f_{5}+1}}; & \;\varphi =0 \end{array}$$

## References

[1] Horodecki R., Horodecki P. (1996). Perfect correlations in the Einstein-Podolsky-Rosen experiment and Bell’s inequalities. Phys. Lett. A.

[2] Einstein A., Podolsky B., Rosen N. (1935). Can Quantum-Mechanical Description of Physical Reality Be Considered Complete?. Phys. Rev..

[3] Bell J.S. (1964). On the Einstein Podolsky Rosen paradox. Phys. Phys. Fiz..

[4] Greenberger D.M., Horne M.A., Shimony A., Zeilinger A. (1990). Bell’s theorem without inequalities. Am. J. Phys..

[5] Schlienz J., Mahler G. (1996). The maximal entangled three-particle state is unique. Phys. Lett. A.

[6] Linden N., Popescu S., Popescu S. (1998). On Multi-Particle Entanglement. Fortschritte Phys..

[7] Horodecki M. (2001). Entanglement measures. Quantum Inf. Comput..

[8] Bennett C.H., DiVincenzo D.P., Smolin J.A., Wootters W.K. (1996). Mixed-state entanglement and quantum error correction. Phys. Rev. A.

[9] Hill S.A., Wootters W.K. (1997). Entanglement of a Pair of Quantum Bits. Phys. Rev. Lett..

[10] Wootters W.K. (1998). Entanglement of Formation of an Arbitrary State of Two Qubits. Phys. Rev. Lett..

[11] Coffman V., Kundu J., Wootters W.K. (2000). Distributed entanglement. Phys. Rev. A.

[12] Osborne T.J., Verstraete F. (2006). General Monogamy Inequality for Bipartite Qubit Entanglement. Phys. Rev. Lett..

[13] Dür W., Vidal G., Cirac J.I. (2000). Three qubits can be entangled in two inequivalent ways. Phys. Rev. A.

[14] Verstraete F., Dehaene J., De Moor B., Verschelde H. (2002). Four qubits can be entangled in nine different ways. Phys. Rev. A.

[15] Acín A., Andrianov A., Costa L., Jané E., Latorre J.I., Tarrach R. (2000). Generalized Schmidt Decomposition and Classification of Three-Quantum-Bit States. Phys. Rev. Lett..

[16] Mosseri R., Dandoloff R. (2001). Geometry of entangled states, Bloch spheres and Hopf fibrations. J. Phys. A Math. Gen..

[17] Mosseri R. (2006). Two-Qubit and Three-Qubit Geometry and Hopf Fibrations. Topology in Condensed Matter.

[18] Sudbery A. (2001). On local invariants of pure three-qubit states. J. Phys. A Math. Gen..

[19] Cayley A. (1843). On the theory of determinants. Transactions of the Cambridge Philosophical Society.

[20] Pérez-Salinas A., García-Martín D., Bravo-Prieto C., Latorre J.I. (2020). Measuring the Tangle of Three-Qubit States. Entropy.

[21] Gelfand I., Kapranov M., Zelevinsky A. (1994). Discriminants, Resultants, and Multidimensional Determinants.

[22] Fano U. (1957). Description of States in Quantum Mechanics by Density Matrix and Operator Techniques. Rev. Mod. Phys..

[23] Walter M., Doran B., Gross D., Christandl M. (2013). Entanglement Polytopes: Multiparticle Entanglement from Single-Particle Information. Science.

[24] Rajwade A.R. (2001). Convex Polyhedra with Regularity Conditions and Hilbert’s Third Problem.

[25] Enríquez M., Delgado F., Życzkowski K. (2018). Entanglement of Three-Qubit Random Pure States. Entropy.

[26] Luna-Hernández S., Enríquez M., Rosas-Ortiz O. (2024). A geometric formulation to measure global and genuine entanglement in three-qubit systems. Sci. Rep..

[27] Vesperini A., Bel-Hadj-Aissa G., Capra L., Franzosi R. (2024). Unveiling the geometric meaning of quantum entanglement: Discrete and continuous variable systems. Front. Phys..

[28] Cocchiarella D., Scali S., Ribisi S., Nardi B., Bel-Hadj-Aissa G., Franzosi R. (2020). Entanglement distance for arbitrary *M*-qudit hybrid systems. Phys. Rev. A.

[29] Haake F. (2001). Level Repulsion. Quantum Signatures of Chaos.

[30] Rosenzweig N., Porter C.E. (1960). “Repulsion of Energy Levels” in Complex Atomic Spectra. Phys. Rev..

[31] Frank W., von Brentano P. (1994). Classical analogy to quantum mechanical level repulsion. Am. J. Phys..

[32] Novotny L. (2010). Strong coupling, energy splitting, and level crossings: A classical perspective. Am. J. Phys..

[33] Ma T., Serota R.A. (2012). Level repulsion in integrable systems. Int. J. Mod. Phys. B.

[34] Briegel H.J., Raussendorf R. (2001). Persistent Entanglement in Arrays of Interacting Particles. Phys. Rev. Lett..

[35] Ghahi M.G., Akhtarshenas S.J. (2016). Entangled graphs: A classification of four-qubit entanglement. Eur. Phys. J. D.

[36] Cervera-Lierta A., Gasull A., Latorre J.I., Sierra G. (2018). Multipartite entanglement in spin chains and the hyperdeterminant. J. Phys. A Math. Theor..

[37] Acín A., Andrianov A., Jané E., Tarrach R. (2001). Three-qubit pure-state canonical forms. J. Phys. A Math. Gen..

[38] Carteret H.A., Higuchi A., Sudbery A. (2000). Multipartite generalization of the Schmidt decomposition. J. Math. Phys..

